# Lack of durable natriuresis and objective decongestion following SGLT2 inhibition in randomized controlled trials of patients with heart failure

**DOI:** 10.1186/s12933-023-01946-w

**Published:** 2023-08-02

**Authors:** Milton Packer

**Affiliations:** Heart and Vascular Institute, Baylor University Medical Center, 621 North Hall Street, Dallas, TX 75226 USA

**Keywords:** Sodium-glucose cotransporter 2 inhibitors, Heart failure, Congestion, Natriuretic peptides

## Abstract

Patients with heart failure have increased cardiac filling pressures, circulating natriuretic peptides, and physical signs of fluid retention, which are related to sodium retention by the kidneys and are alleviated by conventional diuretics. Sodium-glucose cotransporter 2 (SGLT2) inhibitors interfere with sodium and glucose reabsorption in the proximal renal tubule, but they evoke a marked counterregulatory activation of sodium and water reabsorption in distal nephron segments, which opposes and negates any diuretic effect. Nevertheless, it has been postulated that SGLT2 inhibitors modulate the volume set point, leading selectively to decongestion in patients with fluid overload. This hypothesis was tested in a review of 15 randomized controlled trials of SGLT2 inhibitors in patients with heart failure, with 7 trials focusing on urinary volume within the first week, and 8 trials focusing on objective decongestion at 12 weeks. In trials < 1 week, SGLT2 inhibition increased urine volume in the first 24 h, but typically without a change in urinary sodium excretion, and this diuresis was not sustained. In 8 trials of 12 weeks’ duration, none reported alleviation of edema, ascites or pulmonary rales. The 2 trials that evaluated changes in left ventricular filling pressure noted no or small changes (1–2 mm Hg); the two trials that measured interstitial lung water or total blood volume found no effect; and 6 of the 7 trials found no decrease in circulating natriuretic peptides. Therefore, randomized controlled trials do not indicate that SGLT2 inhibitors produce a durable natriuresis or objective decongestion in patients with heart failure.

Patients with acutely decompensated or chronic heart failure experience dyspnea at rest or exertion, which is typically accompanied by increased cardiac filling pressures and circulating levels of natriuretic peptides as well as physical signs of fluid retention (i.e., edema, ascites, pulmonary rales or pleural effusion). Many of these clinical and physiological abnormalities are related to the retention of sodium and water by the kidneys, and accordingly, treatment with loop diuretics or aquaretics are accompanied by symptomatic improvement, a reduction in cardiac filling pressures and natriuretic peptides, and alleviation of physical signs of congestion [1–4]. Increases in right and left ventricular filling pressures can be substantially reduced by loop diuretic therapy (typically by > 10 mm Hg) in patients with or without overt physical signs of fluid retention (i.e., jugular venous distension, ascites or peripheral edema) [4].

## Renal tubular actions of SGLT2 inhibitors

Conventional diuretics and aquaretics act on the loop of Henle and more distal segments of the nephron. In contrast, sodium-glucose cotransporter 2 (SGLT2) inhibitors act in the most proximal segments of the renal tubule (i.e., the S1 and S2 segments) and inhibit not only SGLT2, but also sodium-hydrogen exchanger 3 [5, 6]. This combined action increases tubular sodium, chloride and glucose, but it also evokes a marked counterregulatory activation of sodium and water reabsorption in the loop of Henle and more distal nephron segments, which is related to upregulation of vasopressin, aldosterone, a-ketoglutarate, uromodulin and carbonic anhydrase [7–10]. These compensatory mechanisms oppose and negate the natriuretic and osmotic diuretic response to SGLT2 inhibitors, leading to marked attenuation and truncation of any short-term increase in urine volume or sodium excretion [11, 12]. Nevertheless, it has been postulated that SGLT2 inhibitors modulate the volume set point, [13] leading selectively to a meaningful natriuresis and objective evidence of decongestion in patients with fluid overload, i.e., those with heart failure.

In order to evaluate this possibility, we evaluated all randomized controlled trials of SGLT2 inhibitors in patients with chronic or acutely decompensated heart failure for (1) evidence of a meaningful natriuresis; and (2) objective evidence of decongestion, assessed by physical signs and physiological testing.

## Natriuretic effect of SGLT2 inhibitors in patients with heart failure

The natriuretic effect of drugs is generally assessed by the measurement of urinary sodium excretion. However, urinary sodium measurements can be made on 24-hour collection or on spot urine collections, with the former being subject to less sampling error. Nevertheless, even 24-hour urine collections are influenced by changes in dietary sodium or concomitant medications. These confounding influences are reduced if the data are collected in randomized controlled clinical trials. Body weight is typically used to assess the magnitude of a diuretic effect over short periods of time (e.g., < 1 week), but this metric cannot be used to assess the natriuretic effect of SGLT2 inhibitors over longer periods, because glycosuria causes weight loss through caloric loss in the urine [14]. Furthermore, because SGLT2 inhibitors induce erythropoiesis, increases in hemoglobin and hematocrit cannot be used to infer the occurrence of hemoconcentration and intravascular decongestion, even during treatment periods as short as 7 days [15, 16].

Seven randomized controlled trials have evaluated the effect of SGLT2 inhibitors on urinary sodium and volume in patients with chronic heart failure, Table 1 [17–24]. All patients had elevated levels of natriuretic peptides, implying the high likelihood of elevated cardiac filling pressures and fluid overload. Three trials evaluated patients with stable chronic heart failure, whereas four trials studied patients who had been hospitalized for worsening symptoms of heart failure. Most of the trials evaluated the effect of these drugs over short periods, typically less than 1–4 weeks, and all enrolled fewer than 100 patients.

In patients with acute or chronic heart failure, SGLT2 inhibition produced increases in urine volume in the first 24 h, but typically without a change in urinary sodium excretion. This increase may have been related to an osmotic diuresis secondary to enhanced glycosuria, but the diuresis was generally not sustained beyond 24 h, presumably related to the activation of compensatory mechanisms, i.e., vasopressin [10]. There were no consistent decreases in body weight, circulating natriuretic peptides or dyspnea scores during observation periods of < 1 week. Only one study reported a persistent natriuretic effect accompanied by a decline in body weight after 2 weeks, [17] but interestingly, this study enrolled clinically euvolemic patients who had the lowest levels of circulating natriuretic peptides among all the trials in Table 1. Although some have proposed that SGLT2 inhibitors may potentiate the effects of loop diuretics, the natriuretic potentiation effect with SGLT2 inhibitors (if any) was less marked than with metolazone [24]. There was little evidence that SGLT2 inhibitors were particularly likely to produce a natriuresis in patients with notable fluid overload.

**Table 1:** Effect of SGLT2 Inhibitors on Urinary Sodium Excretion and Objective Evidence of Congestion in Randomized Controlled Trials of Patients With Heart Failure

Study	Patient Population	Baseline Natriuretic Peptides	Study Design	Study Drugs	Major Findings
*Small Short-Term Mechanistic Studies (n < 100)*					
Griffin et al. [17]	20 patients, euvolemic heart failure, diabetes and resistant to loop diuretics	NT-proBNP ≈400 pg/ml	Double-blind, randomized, placebo-controlled crossover trial	Empagliflozin 10 mg/day vs. placebo, each for 14 days	SGLT2i increased fractional sodium excretion after 1 and 14 days. Decrease in body weight and blood volume at 14 days, but no change in NT-proBNP.
Kolwelter et al. [18]	74 patients with stable euvolemic heart failure	NT-proBNP ≈450 pg/ml	Double-blind, randomized, placebo-controlled parallel-group trial	Empagliflozin 10 mg/day vs. placebo for 3 months	SGLT2i increased urinary volume but not urinary sodium excretion at 1 month. No between-group differences in urinary volume, urinary sodium excretion or NT-proBNP after 3 months. No effect on extracellular water at 1 or 3 months.
RECEDE-CHF [19]	23 patients with diabetes and stable heart failure	NT-proBNP ≈2400 pg/ml	Double-blind, randomized, placebo-controlled, crossover trial	Empagliflozin 25 mg/day vs. placebo, each given for 6 weeks	SGLT2i increased urinary volume after 3 days and 6 weeks due to increase in free water clearance. No short-term or long-term changes in 24-hour urinary sodium excretion. No change in NT-proBNP after 3 days or 6 weeks. Weight loss at week 6, but not after 3 days.
EMPAG-HF [20]	60 patients, hospitalized for worsening heart failure	NT-proBNP ≈3400 pg/ml	Double-blind, randomized, placebo-controlled, parallel-group trial	Empagliflozin 25 mg/day vs. placebo for 5 days	SGLT2i increased urine volume over 5 days, but no effect on spot urinary sodium concentration or fractional sodium excretion. Decrease in NT-proBNP but not in body weight after 5 days.
EMPA-RESPONSE-AHF [21, 22]	79 patients, hospitalized for worsening heart failure	NT-proBNP 5200 pg/ml	Double-blind, randomized, placebo-controlled, parallel-group trial	Empagliflozin 10 mg/day or placebo for 30 days	SGLT2i increased urine volume, but no effect on fractional sodium excretion, spot urinary sodium concentration, NT-proBNP, dyspnea score or body weight after 4 days.
Tamaki et al. [23]	59 patients with diabetes, hospitalized for worsening heart failure	NT-proBNP 3200 pg/ml	Open-label randomized parallel-group trial	Empagliflozin 10 mg/day vs. other diabetic therapy for 7 days	SGLT2i increased urinary volume and urinary sodium excretion at 24 h; no assessments at day 7. No change in body weight after 1 or 7 days. Lower NT-proBNP at day 7.
Yeoh et al. [24]	61 patients hospitalized for worsening heart failure and fluid retention, diuretic resistant	NT-proBNP ≈4000 pg/ml	Open-label, randomized, parallel-group trial	Dapagliflozin 10 mg/day vs. metolazone 5–10 mg/day for 3 days	No change in spot urinary sodium concentration with SGLT2i during 3 days. Less natriuresis, lower loop diuretic efficiency and less weight loss with SGLT2i than metolazone. No between-group difference in interstitial lung fluid.
*Multicenter Efficacy Endpoint Trials (Typically n > 100) of 12 Weeks Duration*					
EMPIRE- HF [30–32]	190 patients with stable heart failure, reduced EF	NT-proBNP ≈600 pg/ml	Double-blind, randomized, placebo-controlled parallel-group trial	Empagliflozin 10 mg/day or placebo for 12 weeks	No between-group differences in NT-proBNP, daily activity or KCCQ. In 70 patients, no between-group difference in pulmonary capillary wedge pressure at rest or exercise after 12 weeks. Total blood volume reduced by only 1%.
PRESERVED-EF [33]	324 patients with heart failure, preserved EF	NT-proBNP ≈700 pg/ml	Double-blind, randomized, placebo-controlled parallel-group trial	Dapagliflozin 10 mg/day vs. placebo, for 12 weeks.	SGLT2i improved KCCQ and 6-minute walk distance, without change in NT-proBNP. Body weight decreased by ≈0.8 kg after 12 weeks.
EMBRACE-HF [34]	65 patients with stable heart failure	NT-proBNP ≈800 pg/ml	Double-blind, randomized, placebo-controlled parallel-group trial	Empagliflozin 10 mg/day vs. placebo, for 12 weeks	Pulmonary arterial diastolic pressure was ≈1–2 mm Hg lower in the SGLT2i group, which persisted even when drug withdrawn for 1 week. No between-group differences in 6-minute walk distance, KCCQ or NT-proBNP at 12 weeks
CHIEF-HF [35]	448 patients with stable heart failure	Not performed	Double-blind, randomized, placebo-controlled parallel-group trial	Canagliflozin 100 m/day vs. placebo, for 12 weeks	SGLT2i improved KCCQ scores at 12 weeks. No assessments of congestion or cardiac filling pressures were performed.
EMPERIAL-Preserved [36]	315 patients with stable heart failure, preserved EF	NT-proBNP ≈900 pg/ml	Double-blind, randomized, placebo-controlled parallel-group trial	Empagliflozin 10 mg/day vs. placebo, for 12 weeks	No between-group difference in 6-minute walk distance, dyspnea score, KCCQ scores, clinical congestion score or NT-proBNP at 12 weeks.
DEFINE-HF [37, 38]	263 patients with stable heart failure	NT-proBNP ≈1100 pg/ml	Double-blind, randomized, placebo-controlled parallel-group trial	Dapagliflozin 10 mg/day vs. placebo, for 12 weeks.	SGLT2i improved KCCQ scores at 12 weeks, but did not have an effect on NT-proBNP at 6 or 12 weeks. In subgroup of 85 patients, no change in lung fluid volume at 12 weeks vs. placebo
EMPERIAL-Reduced [36]	312 patients with stable heart failure, reduced EF	NT-proBNP ≈1500 pg/ml	Double-blind, randomized, placebo-controlled parallel-group trial	Empagliflozin 10 mg/day vs. placebo, for 12 weeks	No between-group difference in 6-minute walk distance, and no effect on dyspnea score or NT-proBNP at 12 weeks. Nominally significant between-group difference in KCCQ and clinical congestion score (based on orthopnea)
EMPULSE [39–41]	530 patients hospitalized for worsening heart failure	NT-proBNP ≈3200 pg/ml	Double-blind, randomized, placebo-controlled parallel-group trial	Empagliflozin 10 mg/day vs. placebo, for 90 days	SGLT2i improved KCCQ at 15 and 30 days and decreased NT-proBNP at 30 days. Decrease in clinical congestion score (based on symptoms) at 15 days. Weight loss and increase in hematocrit at 15 days attributed to diuresis and hemoconcentration.

Abbreviations: EF = ejection fraction; KCCQ = Kansas City Cardiomyopathy Questionnaire; NT-proBNP = N-terminal prohormone B-type natriuretic peptide; SGLT2i = sodium-glucose cotransporter 2 inhibition

## Effect of SGLT2 inhibitors on objective signs of decongestion

Many physicians consider dyspnea (at rest or during exertion) as evidence of symptomatic congestion. The effects of dyspnea or effort tolerance and health status can be assessed by formal exercise testing (e.g., a 6-minute walk distance or cardiopulmonary exercise testing) or by the Kansas City Cardiomyopathy Questionnaire (KCCQ), but these assessments may not be concordant [25]. Furthermore, by improving ventricular performance or by effects on the peripheral circulation or skeletal muscle performance, many drugs for heart failure (e.g., beta-blockers and systemic vasodilators) can alleviate dyspnea and improve exercise capacity in the absence of any effect on the kidney to promote urinary sodium and water excretion [26, 27]. Therefore, this review did not regard a reduction in the symptoms of heart failure (e.g., an improvement in KCCQ scores) as providing reliable evidence of decongestion. Instead, the current analysis focused its attention on objective evidence for the alleviation of fluid retention. Objective decongestion was defined as the alleviation of physical signs of fluid retention (i.e., edema, ascites, pulmonary rales or pleural effusion). Whenever available, the assessment of objective decongestion was supported by changes in left ventricular filling pressure or natriuretic peptides, even though it is understood that these variables can improve markedly without an effect of treatment on urinary sodium or water excretion [26–29].

Eight randomized controlled trials have evaluated the effect of SGLT2 inhibitors on symptoms of heart failure, exercise capacity or health status — together with measures of left ventricular filling pressure, natriuretic peptides or objective evidence of congestion — typically in multicenter trials of < 600 patients, studied for 12 weeks [30–41]. Although many of these trials reported an improvement in KCCQ scores, no trial reported alleviation of physical signs of congestion, i.e., edema, ascites, pulmonary rales or pleural effusion. One trial reported a lessening of “clinical congestion” in patients with acutely decompensated heart failure after 15 days, [39–41] but in this trial, clinical congestion was assessed entirely by symptoms, and the investigators did not report changes in physical signs of fluid retention, even though these were apparent at the time of randomization. The two trials that evaluated changes in pulmonary capillary wedge pressure or pulmonary arterial diastolic pressure noted no or small changes (i.e., 1–2 mm Hg) [31, 34]. The one trial that measured total blood volume noted no treatment effect [32]. Six of the seven trials that evaluated changes in circulating natriuretic peptides found no effect of SGLT2 inhibition. One trial assessed changes in lung fluid volume after 12 weeks using noninvasive impedance measurements and found no difference between patients receiving an SGLT2 inhibitor and those receiving placebo [38]. Reported changes in body weight and hematocrit in some trials could be readily attributable to urinary caloric loss [14] (rather than diuresis) or to erythropoiesis [15, 16] (rather than hemoconcentration).

## Conclusions

Conventional diuretics produce a natriuresis that acts to alleviate objective signs of fluid retention in patients with heart failure, thus minimizing jugular venous distension, pulmonary rales, ascites and peripheral edema [1–4]. In contrast, the action of SGLT2 inhibitors in the proximal renal tubules elicits a vigorous counterregulatory response that minimizes any natriuretic or osmotic diuretic effect. As a result, SGLT2 inhibitors have not been shown to produce a durable natriuretic effect or to alleviate objective signs of congestion in randomized controlled trials (Table 1). In these trials, SGLT2 inhibitors did not generally produce meaningful changes in pulmonary wedge pressure, interstitial lung fluid or circulating natriuretic peptides during treatment periods lasting up to 12 weeks. Therefore, the effect of SGLT2 inhibitors to improve health status (i.e., KCCQ scores) and cardiac structure and function as well as decreasing the risk of cardiovascular death or hospitalization for heart failure [42, 43] are likely due to effects that are unrelated to a discernable action of SGLT2 inhibitors in the kidney during the first 3 months of treatment [44]. This conclusion is supported by observations that SGLT2 inhibitors produce cardioprotective effects in isolated cardiomyocytes (that do not express SGLT2) and in animal models in which SGLT2 has been knocked out [44, 45].

## Data Availability

There are no original data in this paper.
